# Electropenetrography Monitoring of the Neotropical Brown-Stink Bug (Hemiptera: Pentatomidae) on Soybean Pods: An Electrical Penetration Graph-Histology Analysis

**DOI:** 10.1093/jisesa/iey108

**Published:** 2018-11-10

**Authors:** Tiago Lucini, Antônio R Panizzi

**Affiliations:** Laboratório de Entomologia, Embrapa Trigo, Caixa Postal, Passo Fundo, RS, Brasil

**Keywords:** Heteroptera, Pentatomidae, electrical penetration graph, electronic feeding monitoring, feeding site

## Abstract

The Neotropical brown-stink bug *Euschistus heros* (F.) (Hemiptera: Pentatomidae) is the most important pest damaging soybean in the Neotropics, the world largest production area. The alternating current–direct current (AC–DC) electropenetrography (EPG) technology was used to develop an EPG waveform library of adult females feeding on soybean (*Glycine max* (L.) Merr.) pods at different input resistor (Ri) levels. Thirteen waveform types/subtypes were identified and described. They were divided into non-probing (Z, Np, Dw1, and Dw2), and probing waveforms (Eh1, Eh2, Eh3, Eh4, and Eh5). Probing waveforms were grouped into three phases: 1) pathway (Eh1a, Eh1b, Eh1c, and Eh1w), 2) ingestion (Eh2, Eh3a, Eh3b, and Eh4), and 3) interruption (Eh5). Correlations between waveforms Eh1b, Eh1c, Eh2, Eh3, and Eh4 and stylets tip position and/or salivary sheath in the pod tissue were determined via histological studies. Non-probing waveforms, Z and Np were visually associated with the bug resting and walking on pod surface, respectively. Waveform Dw1 was correlated with egestion, and the ingestion of fluids (droplets) was proposed for Dw2. Eh1a and Eh1b corresponded to initial and deep stylet penetration through pod tissue, and secretion of a salivary sheath. In Eh1c, stylets penetrated the rigid cell layer of sclerenchyma, and during Eh1w they were withdrawn. Eh2 represents sustained xylem sap ingestion. Eh3a corresponded to lacerate and macerate cell rupture feeding behavior in seed endosperm, whereas Eh3b corresponded to ingestion of cellular contents. Eh4 represented short ingestion from an unknown site, and Eh5 represented short interruptions during xylem sap ingestion.

The Neotropical brown-stink bug *Euschistus heros* (F.) was rarely found on crops in the 1970s in the Neotropical Region (Panizzi et al. 1977); nowadays, it is considered the most relevant pest species damaging soybean (*Glycine max* (L.) Merr.) among all stink bug species found on this crop (Sosa-Gómez et al. 2009, Panizzi et al. 2012). The massive adoption of no-tillage cultivation systems, introduction of multiple cropping, and the expansion of soybean areas are the primary factors that have caused substantial increase in *E. heros* populations, particularly in Brazil (Panizzi 2015). The pest was reported in soybean fields in Argentina (Saluso et al. 2011), and it is also considered a potential stink bug to invade the United States (Panizzi 2015). Moreover, *E. heros* is considered more tolerant to insecticides than others stink bugs commonly found in soybean fields (Willrich et al. 2003, Snodgrass et al. 2005).

In the Neotropics, *E. heros* has preference to feed on fabaceous plants, mostly on soybean (seed endosperm, its preferred food source). It has been observed on other 20 plant species from 10 different families, with preference, besides Fabaceae, for Solanaceae, Brassicaceae and Asteraceae (Smaniotto and Panizzi 2015). Recently, it was found feeding on cotton (*Gossypium hirsutum* L.) plants (Malvaceae) in central-west Brazil, where it is widespread (Soria et al. 2017).

Due to the pest status of *E. heros*, more studies are needed to better understand its feeding behavior to devise potential control techniques. A tool known as electropenetrography (EPG) provides complete and accurate means to evaluate the feeding behavior of any hemipteran (Walker 2000). In this technique, an electrical circuit between the sucking insect and its food is created, and the low electrical current that flows through the system generates waveforms that represent different feeding activities, including stylet penetration, salivation, and ingestion (Tjallingii 1978, Walker 2000).

EPG has been widely applied to evaluate the feeding behavior of aphids (McLean and Kinsey 1964), leafhoppers (Almeida and Backus 2004), psyllids (Bonani et al. 2010), and, more recently, pentatomids (Lucini and Panizzi 2018). The first electropenetrograph generation was designed using alternating current (AC) applied signal and a low input resistor (Ri) (amplifier sensitivities) of 10^6^ Ohms (McLean and Kinsey 1964). In 1970s the second generation of EPG was develop using direct current (DC) applied signal and a higher Ri level, 10^9^ Ohms (Tjallingii 1978); and more recently, the third generation of EPG was develop using both AC and DC applied signals and multiple Ri’s, ranging from 10^6^ to 10^13^ Ohms (Backus and Bennett 2009).

Compared to those old EPG monitors, the new AC–DC electropenetrograph provides several settings to register feeding behavior. An improvement is the ability to use a variable and selectable range of Ri’s, allowing the researcher to switch among them. The use of different Ri levels allows to observe and determine relevant information of a waveform, such as appearance, and electrical component or origin (resistance [R] and electromotive force [emf]), which are essential to propose the biological meanings of them (Backus et al. 2013, Cervantes et al. 2016, Lucini et al. 2016, Lucini and Panizzi 2017a).

Therefore, this study aimed to: 1) monitor and characterize an EPG waveform library produced by *E. heros* adult females feeding on soybean pods at the pod-filling stage (R5) at different Ri levels using the new AC–DC EPG monitor; 2) determine their specific feeding sites and; 3) ascertain the biological meanings of the waveforms based on their electrical characteristics and histological correlations.

## Materials and Methods

### Stink Bug Rearing and Soybean Plants

From January to March 2018, adults of *E. heros* were collected from a soybean field at the Embrapa Trigo (Passo Fundo, RS, Brazil), taken to the Laboratory of Entomology and placed inside plastic rearing cages (25 × 20 × 20 cm) lined with filter paper. Fresh green bean pods, *Phaseolus vulgaris* L., mature soybean seeds, and raw shelled peanuts, *Arachis hypogaea* L., were provide as food source and replaced twice per week. Eggs were collected from the rearing cages and placed inside plastic boxes (11 × 11 × 3.5 cm) with food to raise nymphs to obtain adults to be used in the experiments. A wet cotton placed on a plastic lid (2 cm diameter) was used to provide water. Rearing cages/boxes were kept in a walk-in chamber at 25 ± 1°C, 65 ± 10% RH and photoperiod of 14:10 (L:D) h.

Soybean plants were grown in a greenhouse. Seeds of cv. BRS 5601 RR (Embrapa) were sown weekly in plastic pots (2-liter) containing a mixture of a prepared soil. Potted plants at the R5 stage (pod filling - Fehr et al. 1971) were taken to the laboratory and placed into a Faraday cage to use in all EPG recordings.

### Insect Wiring and EPG Data Acquisition

*E. heros* females were removed from the colony and starved for 15 h before wiring, without acclimation period on soybean pods. Females were then attached to a gold wire (insect electrode; 3 cm long, 0.1 mm in diameter) according to the methodology described by Lucini and Panizzi (2016). A four-channel AC–DC monitor (Backus and Bennett 2009; EPG Technologies, Inc., Gainesville, FL) was used to record all feeding activities under laboratory conditions (25 ± 1°C) and continuous light for 8 h without interruption. Each insect was individually connected (insect electrode) to the EPG probe (head stage amplifier) and placed on a single pod. Another electrode (plant electrode-5 cm long) was inserted into the soil to create the electrical circuit.

Changes in electrical components were acquired and digitalized at a sample rate of 100 Hz per channel (insect) using a WinDaq DI-710 (Dataq Instruments, Akron, OH) linked to HP Pentium notebook with WinDaq Lite software. To avoid inadvertent rectifier fold-over, pre- and post-rectification output signals were simultaneously recorded at individual channels and matched, using the offset function, whenever necessary to retain the native waveform after rectification (Backus and Bennett 2009). The waveforms were distinguished according to the appearance and electrical characteristics (frequency, amplitude, and electrical origin), and were labeled ‘Eh’ from *E. heros* followed by a number to designate type and plus a lower-case letter to designate subtype, as applied to other studies with stink bugs (Lucini and Panizzi 2016, Lucini et al. 2016).

### Experimental Design

The EPG recordings were done applying four different Ri levels: 10^6^, 10^7^, 10^8^, and 10^9^ Ohms, and a standardized voltage of 50 mV AC for all Ri levels. In total, 50 stink bugs were successfully recorded. The use of different Ri levels allows the determination of the primary component or electrical origin of each waveform recorded and in turn allows the biological meaning of the waveform to be identified. The R-component is emphasized at low Ri levels, while at high Ri levels the emf-component is emphasized (Backus and Bennett 2009). To determine the number and duration of the waveforms, 12 recordings (insects) from 10^7^ and 10^9^ Ohms were used. The EPG waveform events were measured using Windaq Waveform Browser (Dataq Instruments, Akron, OH). Four nonsequential EPG variables (Backus et al. 2007) were calculated: NWEI (number of waveform events per insect), WDI (waveform duration per insect), WDEI (waveform duration per event per insect), and PRT (percentage of recording time).

### Plant Tissue Histology

The salivary sheath/stylet tip position in soybean pods (R5 stage) and the probing waveforms recorded were correlated via histological studies, applying the methodology used by Lucini and Panizzi (2016). A set of *E. heros* females was recorded at Ri 10^7^ Ohms, and 50 mV AC current. The EPG monitor was turned off, when a specific waveform was observed on the computer screen, and then, the stylets were carefully severed using an entomological micro-scissor.

The soybean pod carrying the stylets was detached, and it was hand cut into thin sections using a sharp razor blade (Wilkinson Sword, United Kingdom) under a stereomicroscope (Wild Heerbrugg, Model M5A, Switzerland) for preparing the semi-permanent slides. The position of the stylet tips and/or salivary sheath were determined based on 7 specimens for waveform Eh1b, 5 for waveform Eh1c, 8 for Eh2, 5 for Eh3, and 1 for waveform Eh4. Digital images were captured using an Olympus BX50 (Shinjuku, Tokyo, Japan) microscope coupled with a Sony DXC 107A video camera (Minato, Tokyo, Japan) linked to a computer.

## Results

### Overview of EPG Waveforms for *E. heros*

**Fig. 1.**
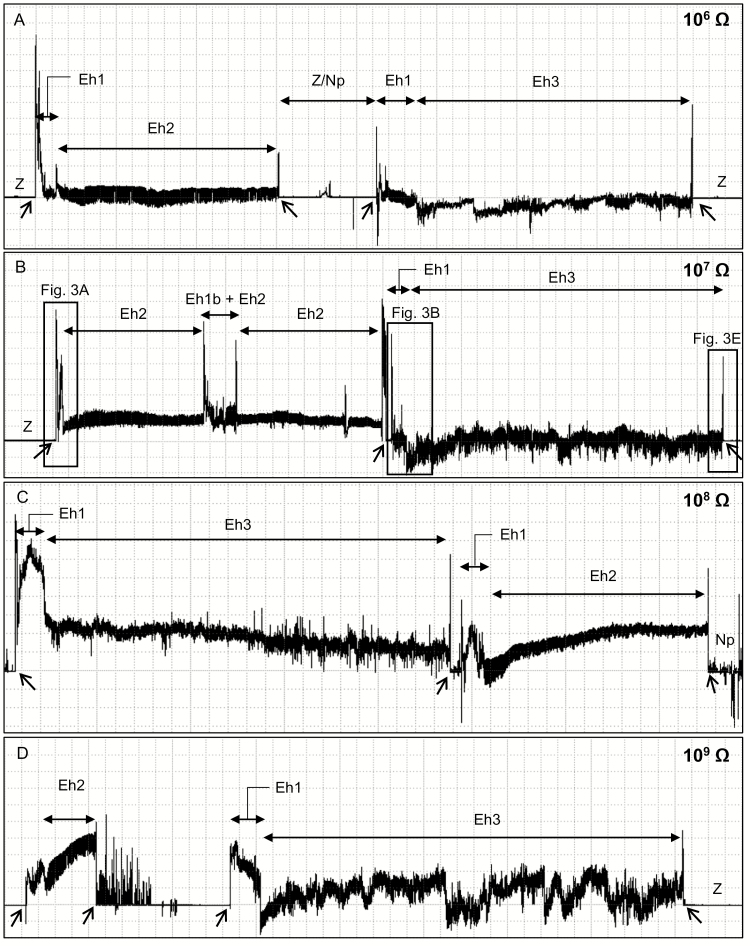
Overview of EPG waveforms produced by *Euschistus heros* on soybean pod (R5 stage [pod filling]) at Ri 10^6^ Ohms (A), 10^7^ Ohms (B), 10^8^ Ohms (C), and 10^9^ Ohms (D) and 50 mV AC applied signal. Coarse structure of waveforms observed with Windaq compression: 800 (160 s/vertical div.), gain 16× (A); 1,500 (300 s/vertical div.), gain 8× (B); 1,300 (260 s/vertical div.), gain 4× (C); 1,500 (300 s/vertical div.), gain 2× (D). Arrowheads indicate beginning or end of a probe.

Thirteen EPG waveform types/subtypes were identified from *E. heros* females placed on soybean pods. These waveforms were described based on their appearance and electrical characteristics (frequency, relative amplitude, and electrical origin), and summarized in Table 1; an overview of the main waveforms recorded at different Ri levels (10^6^ to 10^9^ Ohms) are shown in Fig. 1.

**Table 1.** Summary of EPG AC–DC waveforms, their main characteristics and proposed biological meanings for each waveform recorded during feeding behavior of *Euschistus heros* on soybean pod (at R5 stage [pod filling])

Phase	Family	Type or subtype	Amplitude (%) (range)		Frequency (Hz) (range)		Best seen at Ri levels	Electrical origin	Suggested biological meaning
			Ri 10^7^	Ri 10^9^	Ri 10^7^	Ri 10^9^			
Non-probing	-	Z	Flat	Flat	-	-	10^6^–10^9^	-	Standing still on the plant surface
		Np	Low	Medium-high	-	-	10^8^–10^9^	Mostly emf; some R	Walking on the plant surface
		Dw1	100%	100%	Irregular	Irregular	10^6^–10^8^	Mostly R	Egestion of saliva/regurgitate liquid food on pod surface
		Dw2	10% (6–13)	36% (31–42)	5.6 Hz (5.4–5.8)	4.8 Hz (4.7–5.0)	10^7^–10^9^	emf-dominated	Probably re-ingestion of saliva/regurgitate liquid food
Pathway	P	Eh1a	100%	100%	Irregular	Irregular	10^6^–10^9^	R	Beginning of stylet penetration and secretion of gelling saliva to form salivary sheath
		Eh1b	62% (28–93)	62% (46–91)	Irregular	Irregular	10^6^–10^9^	Mostly R	Deep stylet penetration and secretion of gelling saliva to form branches of a salivary sheath
		Eh1c	43% (28–61)	35% (24–50)	6.5 Hz (5.2–7.5)	6.3 Hz (5.7–6.9)	10^6^–10^9^	R/emf	Bug encountering a rigid cell layer requiring stylet protraction and retraction
		Eh1w	65% (43–100)	65% (43–97)	Irregular	Irregular	10^6^–10^9^	Mostly R	Stylets withdrawal from the plant tissue
Ingestion	I	Eh2	16% (7–31)	33% (24–43)	3.6 Hz (3.2–4.3)	3.8 Hz (2.9–4.4)	10^6^–10^9^	Mixed; peak = R/emf; wave = mostly emf	Sustained xylem sap ingestion
Salivation	I	Eh3a	21% (14–36)	29% (16–47)	Mostly irregular + burst regular sections 3.9 Hz	Mostly irregular + burst regular sections 3.4 Hz	10^6^–10^9^	R/emf	Cell laceration and enzymatic maceration of endosperm tissue
Ingestion	I	Eh3b	8.0% (3–13)	14% (7–30)	4.6 Hz (3.0–5.5)	4.2 Hz (3.5–5.2)	10^7^–10^9^	Mostly emf	Short ingestion event of lacerated/ macerated endosperm tissue
	I	Eh4	4.0% (2–6)	8.0% (5–11)	4.4 Hz (3.2–5.5)	4.4 Hz (3.8–5.4)	Unknown	emf	Short ingestion event from an unknown site
Interruption	N	Eh5	35% (16–70)	29% (17–56)	Irregular	Irregular	10^6^–10^9^	R/emf	Short interruptions during xylem sap ingestion

The waveforms were divided into non-probing waveforms (NP, Z, Dw1, and Dw2), and probing waveforms (Eh1, Eh2, Eh3, Eh4, and Eh5). Probing waveforms were grouped into three main phases: 1) pathway (Eh1), 2) ingestion (Eh2, Eh3, and Eh4), and 3) interruption (Eh5). Pathway phase comprised only one family (labeled - P), which was divided into four different subtypes (Eh1a, Eh1b, Eh1c, and Eh1w). Ingestion phase comprised two distinct families: 1) family I (ingestion [that includes the following waveforms types/subtypes: Eh2, Eh3a, Eh3b, and Eh4]) and 2) family N (interruption [that includes only one waveform type, Eh5]).

### Non-probing Waveforms (Np, Z, Dw1, and Dw2)

Waveform Z appeared as a flat line with very low amplitude across different Ri levels; it represents the baseline of the recording (Fig. 1A, B, and D; Table1). Waveform Np was similar in appearance with several irregular peaks (Fig. 1C) across the different Ri levels. At high Ri levels (10^8^ and 10^9^ Ohms) peaks were greatly emphasized (medium to high amplitude) than at lower Ri levels (10^6^ and 10^7^ Ohms), suggesting a strong emf-component (Table 1).

**Fig. 2.**
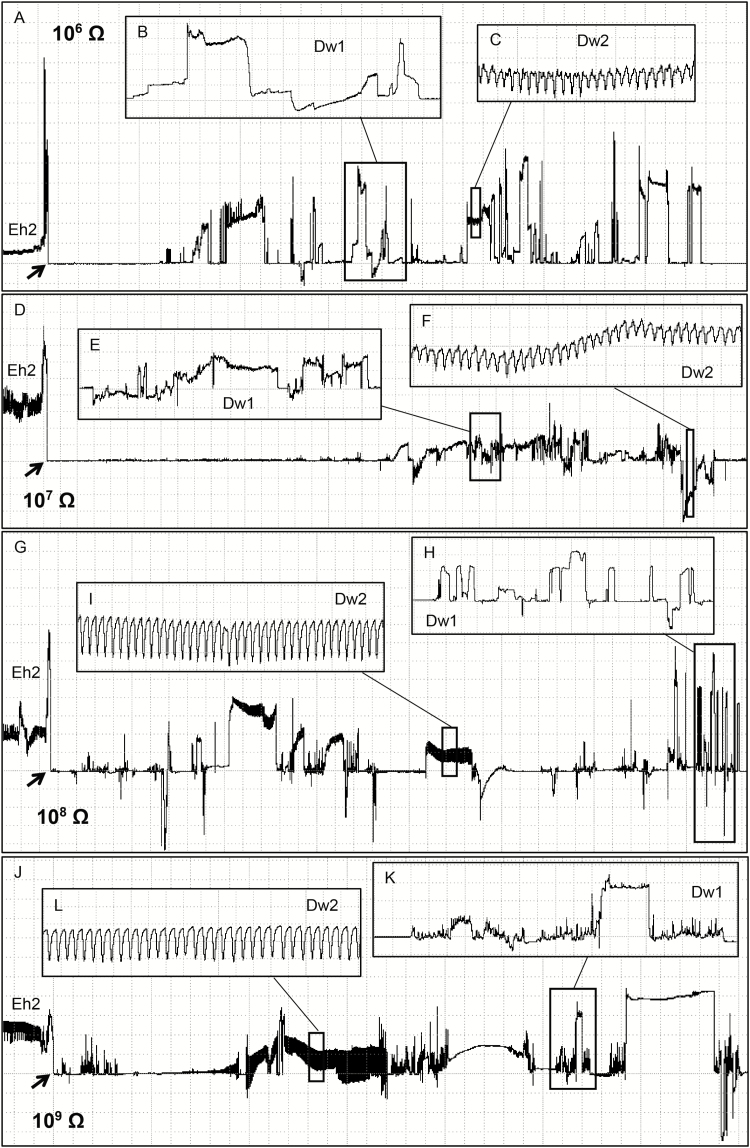
Coarse structure (large boxes) of the food/saliva regurgitate waveforms (Dw1 and Dw2) and their details (small insect boxes) produced by *Euschistus heros* on soybean pod (R5 stage [pod filling]) at Ri 10^6^ Ohms (A–C), 10^7^ Ohms (D–F), 10^8^ Ohms (G–I) and 10^9^ Ohms (J–L) and 50 mV AC applied signal. Overview of the waveforms Dw1 and Dw2 occurring soon after a xylem feeding event (Eh2 wave) (A, D, G, J). Details of the waveform Dw1 (B, E, H, K). Details of the waveform Dw2 (C, F, I, L). Large boxes with Windaq compression 100 (20 s/vertical div.) and gain: 16× (A, D); 4× (G); and 2× (J). Small insect boxes (Dw1 wave) with Windaq compression 10 (2 s/vertical div.) and gain: 16× (B, E); and 2× (H, K). Small insect boxes (Dw2 wave) with Windaq compression 2 (0.4 s/vertical div.) and gain: 128× (C); 64× (F); 16× (I); and 2× (L). Arrowheads indicate the beginning or end of a probe.

Waveform Dw1 occurred after Z or Np, with large and irregular peaks with a high amplitude plateau (Fig. 2A, B, and H; Table 1). At Ri 10^6^ Ohms, Dw1 was clearly visible and distinguished from other waveforms (Fig. 2A); however, as Ri levels increased, Dw1 became less clear (large boxes comparison in Fig. 2), suggesting that Dw1 is R-dominated. Dw2 was a high and regular waveform (4.8–5.6 Hz). It presented a very low amplitude at low Ri levels (10^6^–10^7^ Ohms) and medium amplitude at high Ri levels (mostly 10^9^ Ohms) (Table 1); it means that, as Ri levels increased, the waveform appearance became more visible (Fig. 2A and J), suggesting Dw2 as emf-dominated. Among all waveforms recorded on soybean pods, *E. heros* spent most of the recording time (ca. 57%) on non-probing activities (Table 2).

**Table 2.** EPG nonsequential variables of *Euschistus heros* on soybean pod (R5 stage [pod filling])

Waveform	NWEI	WDI	WDEI	PRT (%)	Proposed activities
Z + Np	-	36.3 ± 8.3	272.4 ± 31.5	56.8	Rest/walking
Dw1	3.0 ± 0.0	5.0 ± 1.1	1.7 ± 0.4	0.3	Egestion of food/saliva
Dw2	2.2 ± 1.0	2.8 ± 2.0	1.3 ± 0.3	0.3	Re-ingestion
Eh1a	5.2 ± 0.6	1.7 ± 0.2	0.2 ± 0.0	0.4	Pathway activities
Eh1b	6.4 ± 0.8	8.8 ± 1.5	1.4 ± 0.1	1.8	
Eh1c	4.2 ± 0.5	20.4 ± 3.5	4.9 ± 0.4	4.3	
Eh1w	4.8 ± 0.5	0.8 ± 0.2	0.2 ± 0.0	0.2	
Eh2	2.7 ± 06	107.7 ± 40.6	39.9 ± 11.6	18.7	Xylem sap ingestion
Eh3a	17.4 ± 3.7	79.8 ± 16.9	4.4 ± 0.4	16.6	Endosperm activities
Eh3b	14.3 ± 3.8	2.2 ± 0.7	0.2 ± 0.0	0.5	
Eh4	2.6 ± 0.2	2.9 ± 0.6	1.1 ± 0.2	0.3	Ingestion from unknown site

NWEI = number (± SE) of waveform events per insect; WDI = waveform duration per insect (min ± SE); WDEI = waveform duration per event per insect (min ± SE), and PRT = percentage of recording time.

### Probing Waveforms: Pathway Phase—Family P (Eh1)

**Fig. 3.**
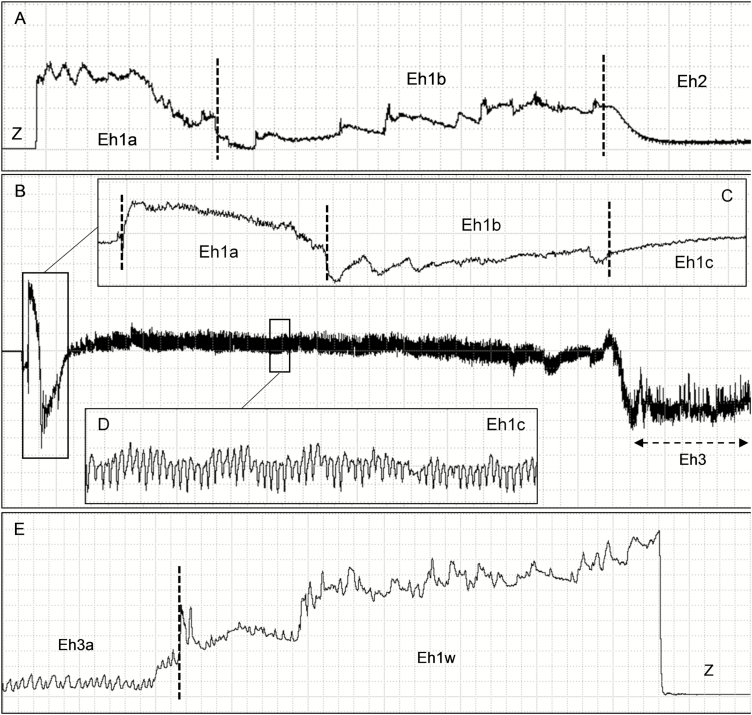
Pathway waveforms (Eh1) produced by *Euschistus heros* on soybean pod (R5 stage [pod filling]) at Ri 10^7^ Ohms and 50 mV AC applied signal. Expanded view of the waveforms Eh1a and Eh1b preceding Eh2 (A). Expanded view of the waveforms Eh1a, Eh1b, and Eh1c preceding Eh3 (B–D). Expanded view of the waveform Eh1w at the end of waveform Eh3 and before Z wave (non-probing) (E). Windaq compression: 20 (4 s/vertical div.), gain 4× (A); 80 (16 s/vertical div.), gain 16× (B); 5 (1 s/vertical div.), gain 8× (C); 2 (0.4 s/vertical div.), gain 32× (D); 2 (0.4 s/vertical div.), gain 16× (E).

This family consisted of only one waveform type Eh1, divided into four subtypes Eh1a, Eh1b, Eh1c, and Eh1w (Table 1; Fig. 3). The first three subtypes represent the initial stylet insertion and deep penetration into the pod tissue, whereas Eh1w represents stylets withdraw at the end of a probing event. The pathway phase, represents ca. 7% of the total recording time (Table 2).

#### Subtype Eh1a

This waveform was visually correlated with rostrum touching the plant surface and beginning stylet insertion into the pod tissue (first probing activity). Eh1a is a brief waveform (WDEI = ~13 s) (Table 2) with irregular peaks randomly distributed and with the highest relative amplitude among all the waveforms registered. Eh1a also presented a high voltage level at the beginning of stylet insertion, which decreased gradually as stylets penetrated deeper into the pod tissue (Fig. 3A–C). Eh1a was greatly emphasized in recordings at Ri 10^6^–10^7^ Ohms, indicating R-component as electrical origin (Table 1).

#### Subtype Eh1b

Eh1b was recorded after Eh1a, and it was much longer (WDEI = 1.4 min) (Table 2). In general, Eh1b appeared as an underlying waveform with continuous increase in voltage level (Fig. 3A–C). Sometimes it presented a stereotypical pattern of episodes, composed by sudden increase in voltage followed by gradual decrease. Electrically, Eh1b presented high amplitude (62%) at both Ri 10^7^ and 10^9^ Ohms, irregular-frequency, and mostly R-component as electrical origin (Table 1).

#### Subtype Eh1c

This waveform was always recorded after Eh1b and presented the longest waveform duration among all pathway waveforms (WDEI ~ 5 min) (Table 2). In some cases, at the beginning random spikes were observed but they disappeared as the waveform became stable. Different from former pathway waveforms, Eh1c was highly regular (5.2 to 7.5 Hz) with downward peaks (Fig. 3D), and medium to high amplitude (from 24 to 61%) among Ri levels applied. Eh1c presented both R and emf components as electrical origin, since it was greatly emphasized at Ri 10^6^ Ohms, and still distinguishable at higher Ri levels (10^8^–10^9^ Ohms) (Table 1). Eh1c occurred sometimes before Eh2 and always before Eh3 (Fig. 3B).

#### Subtype Eh1w

This waveform was recorded always at the end of a probing event and before non-probing waveforms (Z or Np). Similar to Eh1a, Eh1w was very brief (WDEI = ~ 13 s) (Table 2); however, different from Eh1a, it presented an initial low voltage level which gradually increased as the stylets were pulled out from the pod tissue (Fig. 3E). It presented high amplitude (65%) at both Ri 10^7^ and 10^9^ Ohms, irregular-frequency, and mostly R-component (Table 1).

### Probing Waveforms: Ingestion Phase—Family I (Eh2, Eh3, and Eh4)

This family comprised waveforms Eh2, Eh3, and Eh4, with Eh3 divided into two subtypes Eh3a and Eh3b (Table 1). Eh2 and probably Eh4 represent ingestion from vascular vessels, whereas Eh3 represents feeding (preparation and ingestion) from seed endosperm. *E. heros* spent ca. 36% of the recording time at the ingestion phase (Table 2).

#### Type Eh2

**Fig. 4.**
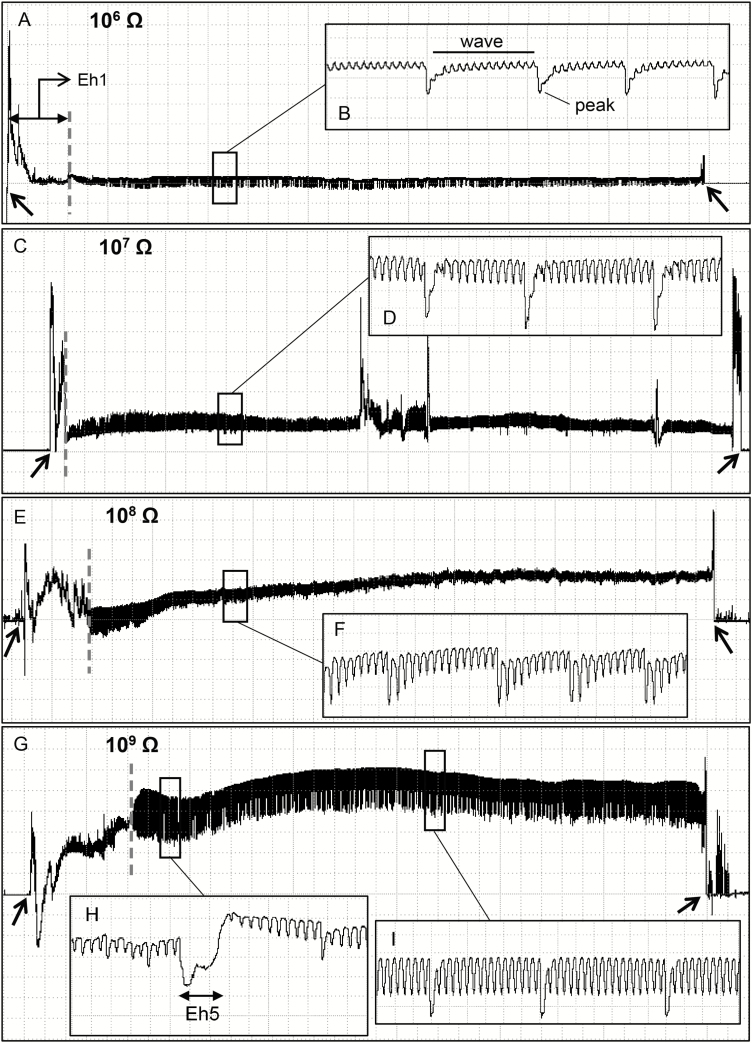
Coarse structure (large boxes) of the xylem waveforms (Eh2) and their details (small insect boxes) produced by *Euschistus heros* on soybean pod (R5 stage [pod filling]) at Ri 10^6^ Ohms (A, B), 10^7^ Ohms (C, D), 10^8^ Ohms (E, F), and 10^9^ Ohms (G–I) and 50 mV AC applied signal. Definition of waves and peaks portions (B). Waveform Eh5 occurring during Eh2 wave (H). Large boxes with Windaq compression: 350 (70 s/vertical div.), gain 8× (A); 800 (160 s/vertical div.), gain 8× (C); 500 (100 s/vertical div.), gain 4× (E); 300 (60 s/vertical div.), gain 4× (G). Small insect boxes (details) with Windaq compression 3 (0.6 s/vertical div.) and gain: 32× (B, D); 16× (F); and 8× (H, I). Arrowheads indicate the beginning or end of a probe.

Eh2 was often preceded by Eh1b or sometimes by Eh1c. It shows regular wave portions (~3.7 Hz) interspersed with peaks regularly distributed and mostly downward oriented (Fig. 4A–I), although in some xylem events they might be upward oriented (this inversion was not due to rectifier fold-over, see Materials and Methods section).

The electrical analysis indicates that Eh2 has a mixture of R and emf components. At low Ri levels (10^6^ Ohms), peaks were greatly emphasized compared to waves (Fig. 4B), and still visible at high Ri levels (10^8^–10^9^ Ohms) (Fig. 4F and I); this result suggests that peaks present a mixed of R and emf components. As Ri levels increased from 10^6^ to 10^9^ Ohms, the wave portion gained amplitude (10^7^ = 16%; 10^9^ = 33%) (Table 1). Amplitude was tiny at low Ri levels (Fig. 4B and I) suggesting the wave portion is mostly emf-dominated. From all waveforms recorded on soybean pods, Eh2 was the second longest (19% of the recording time). Ingestion events were repeated ca. 3 times per bug (NWEI), duration of ingestion per insect (WDI) was longer (ca. 108 min) compared to the other waveforms, with each ingestion event (WDEI) taking ca. 40 min (Table 2).

#### Type Eh3

**Fig. 5.**
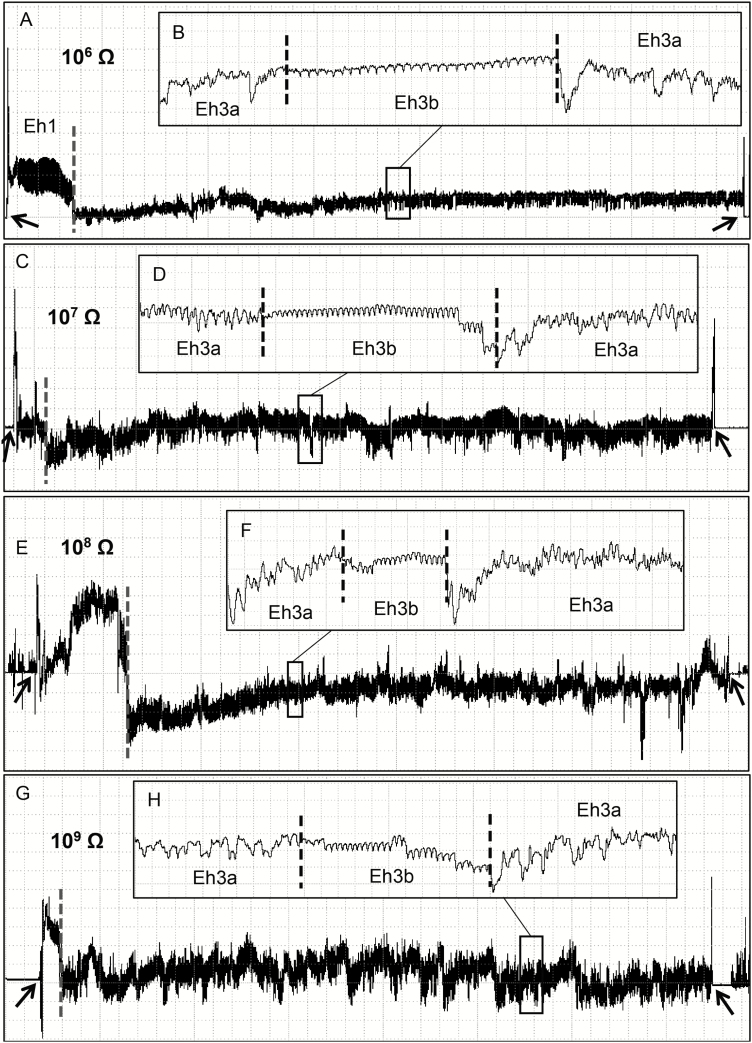
Coarse structure (large boxes) of the endosperm waveforms (Eh3a and Eh3b) and their details (small insect boxes) produced by *Euschistus heros* on soybean pod (R5 stage [pod filling]) at Ri 10^6^ Ohms (A, B), 10^7^ Ohms (C, D), 10^8^ Ohms (E, F), and 10^9^ Ohms (G, H) and 50 mV AC applied signal. Large boxes with Windaq compression: 400 (80 s/vertical div.), gain 16× (A); 800 (160 s/vertical div.), gain 8× (C); 500 (100 s/vertical div.), gain 4× (E); 1,400 (280 s/vertical div.), gain 2× (G). Small insect boxes (details) with Windaq compression 3 (0.6 s/vertical) and gain: 64× (B); 16× (D, F); and 8× (H). Arrowheads indicate the beginning or end of a probe.

Eh3 was always recorded after Eh1c waveform. In general, the transition between the two waveforms were marked by a sudden drop in voltage (Figs. 3B; 5A, C, E, and G). Eh3 was divided into subtypes Eh3a and Eh3b that occurred interspersed (Fig. 5A–H). Eh3a is an irregular waveform with downward peaks (sometimes positive and negative oriented within the same recording, this inversion was not due to rectifier fold-over) irregularly distributed (although with burst of regular sections [3.4–3.9 Hz]), and with low-to-medium amplitude (14–47%) (Table 1). Eh3a was variable in appearance between Ri levels, among individuals, and also within the same recording of an individual. Eh3a was the third longest waveform (ca. 17% of recording time); each event was repeated over 17 times per bug, with total duration per insect of 80 min, with each event taking ca. 4 min (Table 2). As Ri levels increased from 10^6^ to 10^9^ Ohms, Eh3a kept clear appearance (Fig. 5B, D, F, and H) and low-to-medium amplitude values (10^7^ = 21%; 10^9^ = 29%), indicating a mixed of R and emf components.

Eh3b is highly regular (~4.4 Hz) with low amplitude (8–14%). Each event was repeated over 14 times per insect, shorter (~13 s per event) than those at Eh3a wave (Table 2). Eh3b was tiny at 10^6^ Ohms (sometimes not clearly distinguished within recording) and clearly viewed at higher Ri levels (Fig. 5B, F, and H). Therefore, Eh3b is mostly emf-dominated.

#### Type Eh4

**Fig. 6.**
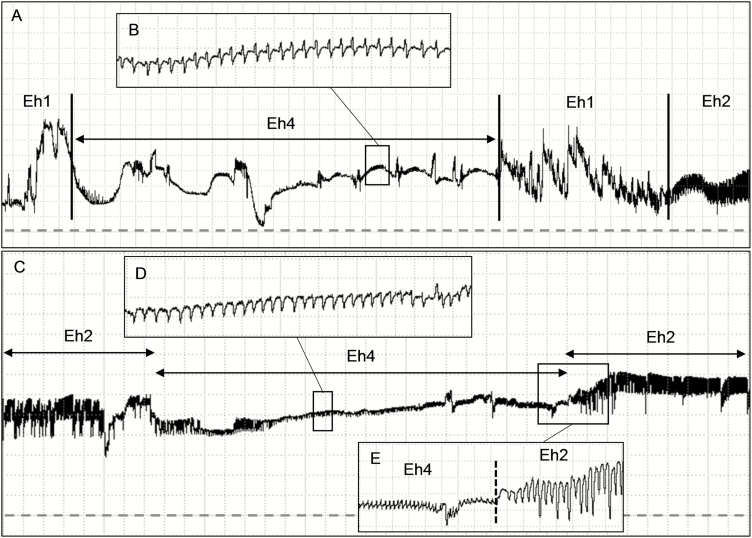
Coarse structure (large boxes) of the unknown ingestion waveform (Eh4) and their details (small insect boxes) produced by *Euschistus heros* on soybean pod (R5 stage [pod filling]) at Ri 10^9^ Ohms and 50 mV AC applied signal. Occurrence of an Eh4 event between two Eh1 events before stink bug reaches the xylem vessels (A). Detail of the waveform Eh4 (B, D). Occurrence of an Eh4 event between two Eh2 events (C). Transition between Eh4 and Eh2 waves (E). Boxes with Windaq compression: 50 (10 s/vertical), gain 4× (A); 3 (0.6 s/vertical div.), gain 16× (B, D); 20 (4 s/vertical div.), gain 4× (C); 5 (1 s/vertical div.), gain 8× (D). Dashed line represents the baseline.

Eh4 was often recorded either between Eh1b events (Fig. 6A) or before/ between Eh2 event(s) (Fig. 6C and E) at all Ri levels applied. Similar to Eh2, Eh4 was a stereotypical and highly regular waveform (Fig. 6B and D), but, in general, without peaks. It shows very low amplitude through Ri levels (4 and 8% at 10^7^ and 10^9^ Ohms, respectively), with sudden decrease between Eh1 or Eh2 events (Fig. 6A and C), regular frequency (4.4 Hz), and emf-component as electrical origin (Table 1). Individuals that produced Eh4 repeated each event ca. three times with short duration per event (~1 min) (Table 2).

### Probing Waveforms: Interruption Phase—Family N (Eh5)

Only one waveform, Eh5, was observed in this family. It represented a short interruption of a few seconds within waveform Eh2 (Fig. 4H), but was not observed for all individuals. It showed an irregular form (flat-spikey plateau) negative or positive oriented, medium amplitude (29–35%) (Table 1), and it occurred mostly at the beginning of Eh2.

### Correlations Between Waveforms and Salivary Sheath/Stylet Tips Position in the Pod Tissue

**Fig. 7.**
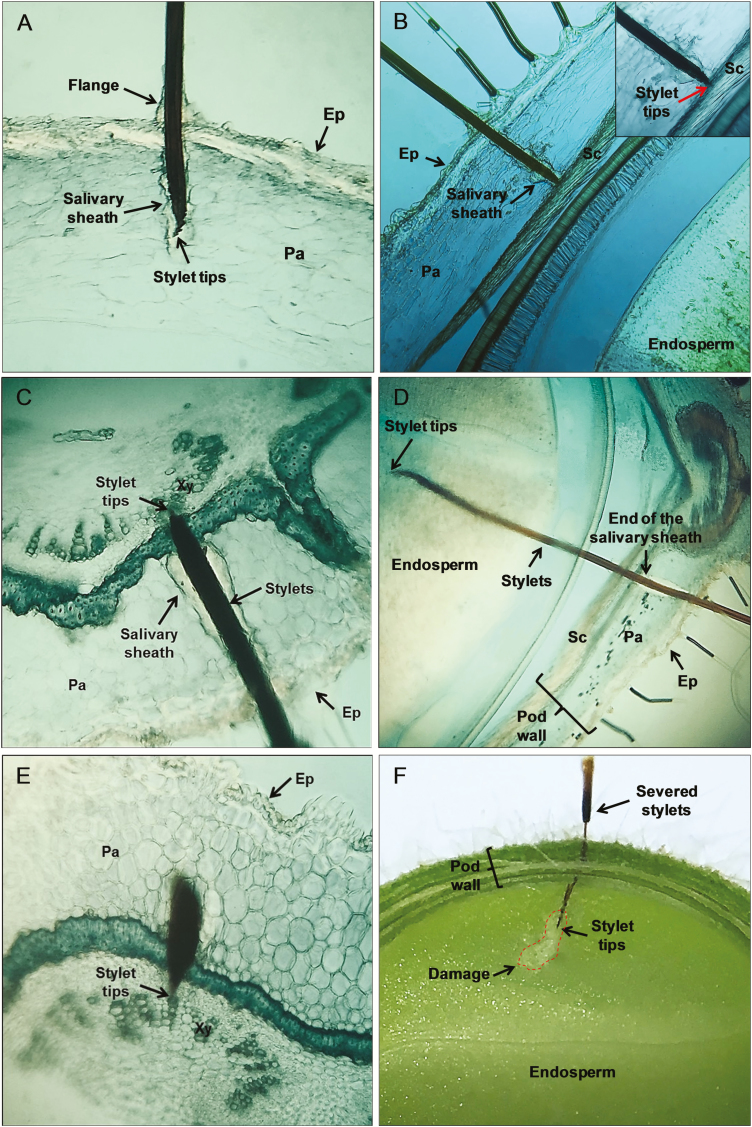
Cross-sections of soybean pods (R5 stage [pod filling]) containing severed stylets and salivary sheath of *Euschistus heros*. Salivary sheath and stylet tips ending in the parenchyma tissue during waveform Eh1b (A). Stylet tips ending in the sclerenchyma layer during waveform Eh1c (indicated by the red arrow in the detail box) (B). Complete salivary sheath and stylet tips ending in the xylem vessels during waveform Eh2 (C). Secretion of an incomplete salivary sheath and stylet tips positioned in endosperm tissue during waveform Eh3 (D). Stylet tips positioned near of xylem vessels during waveform Eh4 (E). Cross-sections of fresh soybean pod containing severed stylets and showing a visual damaged area (opaque region surrounded by the red dashed line) after an Eh3 event (F). Ep = epidermis, Pa = parenchyma, Xy = xylem, Sc = sclerenchyma.

The stylets penetration in the soybean pod tissue and EPG waveforms, coupled with histological analyses, revealed the presence of a salivary sheath surrounding the stylets in the pathway phase. Both stylets and salivary sheath tips were positioned in the parenchyma tissue during waveform Eh1b (*n* = 7) (Fig. 7A), and in the sclerenchyma layer tissue during waveform Eh1c (*n* = 5) (Fig. 7B). When stylets were severed during Eh2, the histological sections (*n* = 8) showed a complete salivary sheath and stylet tips positioned inside the xylem cells (Fig. 7C). For waveform Eh3, an incomplete salivary sheath was observed at the beginning of the stylet penetration (which was secreted during pathway phase) and the stylets tips (*n* = 5) were always positioned in the seed endosperm (Fig. 7D). During waveform Eh4, one histological section obtained showed the stylets positioned near xylem vessels (Fig. 7E). Sections of the fresh soybean pods during Eh3 event revealed a damaged area in the seed endosperm (opaque white region delimited by the dashed red line) (Fig. 7F).

## Discussion

The new AC–DC EPG monitor has allowed researchers to create comprehensive waveform libraries with much more details and accurate information compared to those generated by older monitors (e.g., Backus et al. 2013, Pearson et al. 2014, Lucini et al. 2016, Cervantes and Backus 2018). In this study, we used this new monitor to assess and create a waveform library for *E. heros*, an important pest of soybean in the Neotropics. We have described 13 waveforms related with non-probing and probing behaviors on soybean pods at pod-filling R5 stage. In general, the waveforms recorded for *E. heros* on soybean pods were similar in appearance and biological meanings with those recorded for another important soybean pest, *Piezodorus guildinii* Westwood on the same food (Lucini et al. 2016); however, some peculiarities were observed for *E. heros*.

### Non-probing Waveforms (Z, Np, Dw1, and Dw2)

**Fig. 8.**
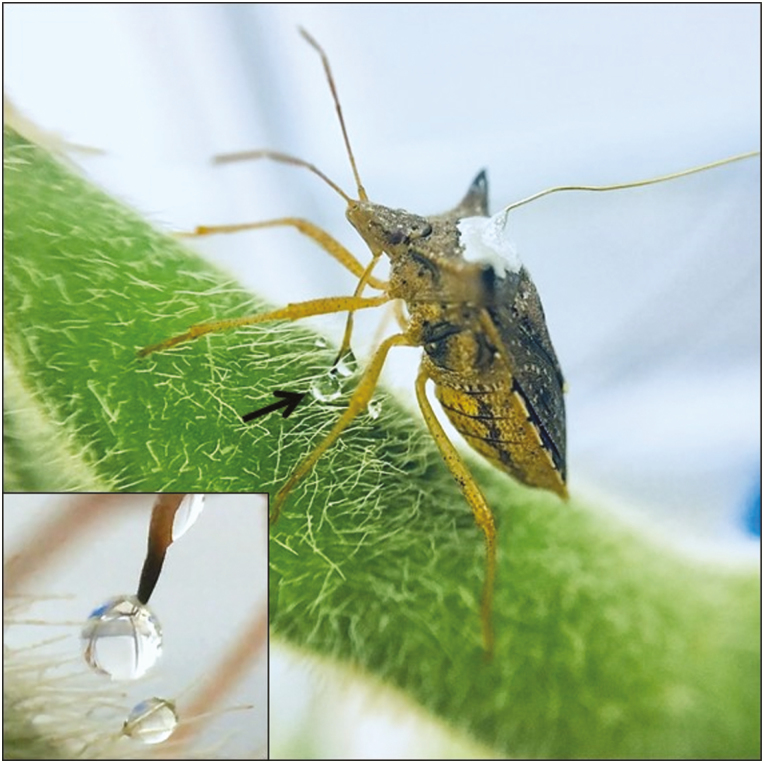
Excretory saliva/regurgitate liquid food (droplets) out of the tip of the stylets produced by *Euschistus heros* adults feeding on soybean pod (R5 stage [pod filling]).

Non-probing waveforms were recorded at all Ri levels applied and were visually characterized and correlated with their biological meaning. During waveform Z the bug was standing still on the plant surface, whereas, waveform Np was associated with the bug walking on the pod surface. At this phase, a curious behavior was observed for *E. heros* (in 35% of the bugs recorded), which, to our knowledge, has not been previously reported in any EPG study; this behavior comprised two distinct waveforms (Dw1 and Dw2). During waveform Dw1, the bugs produced a droplet on the rostrum tip and deposited it on the pod surface (Fig. 8); sometimes, the bug dragged the droplet around without inserting the stylets into the plant tissue. This behavior was mostly recorded soon after a long xylem sap ingestion. Thus, we suspect that the liquid egested might include saliva and/or regurgitation of the excess of liquid taken.

The second waveform recorded (Dw2) occurred when the rostrum tip was inserted in the liquid droplet. Because of its similarity with ingestion waveforms, we suspected it represents the re-ingestion of liquid. For the several species of pentatomids recorded via EPG (see Lucini and Panizzi 2018 and references therein), although sap was ingested from vascular vessels (xylem and/or phloem), none showed this behavior. In one occasion, this behavior was observed for *Dichelops melacanthus* (Dallas) feeding on maize seedlings (Lucini and Panizzi 2017c), but the bug was not connected to the EPG monitor. Clearly, more studies (e.g., analysis of the chemical composition of the droplets) are needed to explain this uncommon behavior performed by *E. heros*.

### Probing Waveforms (Eh1, Eh2, Eh3, Eh4, and Eh5)

Phytophagous hemipterans use two main feeding strategies namely: salivary sheath feeding and cell rupture feeding (Backus et al. 2005b). In the first strategy, gelling saliva is secreted by the bugs to form a complete salivary sheath (i.e., the sheath surrounds the stylets on the entire length toward the ingestion cell). During the cell rupture strategy, two distinct tactics are used, lacerate-and-flush and macerate-and-flush. During laceration, the bug moves its stylets continuously in and out the plant tissue to break a pocket of cells (mechanical action) for after ingestion. During maceration, the bug secrets a watery saliva rich in enzymes to destroy the cells (chemical action) to ingest the degraded cell content.

As reported in studies with EPG, stink bugs use the salivary sheath strategy when feeding on xylem and phloem vessels, and the cell rupture strategy when feeding on seed endosperm (using both lacerate and macerate-and-flush tactics simultaneously) (see details in Lucini and Panizzi 2018). *E. heros* also used salivary sheath strategy feeding on xylem vessels and cell rupture strategy on seed endosperm, but curiously, both strategies were employed on the soybean pods. This ability to switch feeding strategy while feeding on the same food source has been reported to other pentatomids, such as *D. melacanthus* on maize seedlings (Lucini and Panizzi 2017a).

#### Pathway Phase: Initial and Deep Stylet Penetration (Eh1a, Eh1b, Eh1c, Eh1w)

This phase comprised irregular waveforms (except Eh1c) at the beginning of stylets insertion and at the end of a probing event, for all insects recorded at all Ri levels. The first one, Eh1a, was a very short waveform (few seconds) and it was visually correlated to the first contact and initial insertion of the bug’s mouthparts into the pod tissue. During Eh1a the bug secrets the gelling saliva to form the salivary sheath.

At the sequence of the pathway phase, waveform Eh1b is recorded, which is longer than Eh1a and occurred always before Eh1c or Eh2 waves. Eh1b represents stylet penetration deeper into the plant tissue and secretion of gelling saliva forming branches of the salivary sheath as shown in the histological images (Fig. 7A). Eh1a and Eh1b waveforms resemble, in parts, wave H of *Blissus* spp. (Backus et al. 2013), wave B1 of *Homalodisca coagulata* (Say) (Backus et al. 2005a), and waves Pg1a and Pg1c of *P. guildinii* (Lucini et al. 2016). These waves were associated with protracting and retracting of stylets in the tissue, and formation of the salivary sheath; they are strongly R-dominated as saliva is highly electrical conductive.

Eh1c is stereotypical waveform, and different from others pathway waveforms. It showed a high regular pattern in all Ri levels applied. Eh1c was frequently observed before the bugs reached the seed endosperm (waveform Eh3), but sometimes it was recorded preceding xylem sap ingestion (waveform Eh2). This suggest that Eh1c is not an X-wave, a species-specific transition waveform, correlated with stylet penetration and subsequent activities inside the preferred ingestion site (xylem and phloem) (Backus et al. 2009). During the occurrence of this waveform, we observed the bugs pushing down their heads and forcing the stylets into the pod tissue, and then retracting the head and stylets upward, constantly repeating this behavior.

Eh1c waveform is similar in appearance, electrical characteristics and biological meaning with wave Pg1d of *P. guildinii* on soybean pod (Lucini et al. 2016) and Df1b of *Dichelops furcatus* (F.) on wheat seed head (Lucini and Panizzi 2017b). For these last two species, heads pushed down to force stylets into the food to overcome a rigid cell layer was speculated, but no evidence was found to support this. However, histological sections made during Eh1c of *E. heros*, clearly showed the stylets positioned in a rigid cell layer (sclerenchyma cells), which they passed to reach the seed endosperm.

At the end of probing, followed by non-probing phase (Z or Np), a short waveform was recorded, Eh1w. This was associated with *E. heros* quickly retracting their heads and stylets upward to completely withdrawn the stylets from the pod tissue. During this behavior, the salivary sheath, which was deposited during the initial pathway phase, was probably filled, as reported to occur in sharpshooters (Backus et al. 2009, Miranda et al. 2009); however, no evidence was found to support that assumption.

#### Ingestion Phase: Xylem Sap Ingestion (Eh2)

Eh2 waveform is strongly associated with sustained sap ingestion from xylem vessels, because: 1) histological images demonstrated that stylet tips and the complete salivary sheath ended in xylem cells, and 2) the high amplitude of the waveform (ingestion waveforms are emf-dominated, and greatly emphasized at high Ri levels). In xylem-ingesting sharpshooters, the waveform amplitude is proportional to the height of the cibarial uplift of the diaphragm (Dugravot et al. 2008); for *E. heros*, the attempt to overcome the high negative pressure of xylem, caused increase of the waveform amplitude. In addition, Eh2 strongly resembles in appearance and electrical characteristics (high amplitude and mixture of electrical origins, with peaks R-dominated and waves emf-dominated) xylem waveforms recorded for other species of pentatomids, such as wave Pg2 of *P. guildinii* on soybean (Lucini et al. 2016) and Df2 of *D. furcatus* on wheat plants (Lucini and Panizzi 2017b).

Female *E. heros* ingested xylem sap more than twice per recording time, on soybean pod. This is similar to that observed with *D. furcatus* females on immature wheat seed head (Lucini and Panizzi 2017b), but different from *P. guildinii* on soybean pod, where the majority of females ingested xylem sap only once (Lucini et al. 2016). The ingestion of water from xylem vessels aims to maintain body hydration (Spiller et al. 1990), which probably occurs with *E. heros*. Since *E. heros*, *P. guildinii* and *D. furcatus*, are known to be seed-feeders, the ingestion from xylem vessels from reproductive structures may aim to maintain nutrient balance after ingesting from seed endosperm (Lucini et al. 2016), which has low water content (Taiz and Zeiger 2004).

#### Ingestion Phase: Seed Endosperm Activities (Eh3a and Eh3b)

During recordings on pods of soybean, two distinct waveform subtypes were recorded interspersed and clearly separated from each other, Eh3a and Eh3b. Eh3a was visually correlated with *E. heros* moving continuously and deeply its stylets back and forth into the pod tissue (seed endosperm); this behavior represents the cell rupture feeding strategy where the lacerate- and macerate-and-flush tactics were used simultaneously (Lucini and Panizzi 2018). This action dissolves mechanically (laceration) and chemically (maceration-digestive enzymes secreted via watery saliva) a pocket of cells in the seed endosperm allowing ingestion of the degraded cell contents.

Other pentatomid species also use the cell rupture feeding strategy in seed endosperm such as *P. guildinii* on soybean pod (waveform Pg3a - Lucini et al. 2016) and *D. furcatus* on wheat seed head (waveform Df3a - Lucini and Panizzi 2017b). In addition, all those waveforms share similar electrical characteristics and appearance.

The histological images during waveform Eh3 showed the stylet tips positioned in the seed endosperm. In contrast to waveform Eh2, an incomplete salivary sheath was secreted surrounding the stylets at the beginning of the stylet insertion only (during pathway phase). The continuous laceration/maceration activities caused damage in seed endosperm, as observed in cuts of a fresh soybean pod after an Eh3 event. To provide evidence that the Eh3 wave was related to seed activities, in a separate set of recordings, we carefully removed the pod-wall to expose the immature seeds and let the bug feed. Results indicated no pathway waveforms, and the Eh3 waveform was similar to the one recorded from intact pods, where the bugs showed similar behavior (moving stylets in and out during subtype Eh3a).

During laceration/maceration, *E. heros* females do not ingest the cell contents (or might occasionally do), and these activities are probably related to food preparation, via mechanical and chemical actions, for later ingestion of the degraded cell contents. Interspersed between Eh3a events, the waveform subtype Eh3b occurred with the stylets visually observed to be briefly motionless inside the tissue (ca. 9 s per event [WDEI]), and then, the bug moved them again (Eh3a). We suggest that during Eh3b the bug ingests the degraded cell contents previously degraded in Eh3a. This behavior was repeated over the entire waveform. This result is supported by the resemblance of Eh3b with other ingestion waveforms and electrical characteristics, mostly electrical origin, with the wave emphasized on high Ri levels (10^8^ and 10^9^ Ohms), indicating emf-dominance.

In summary, *E. heros* spent about 97% of the time in the seed endosperm with lacerate/macerate tactics (~80 min per insect, WDI) and only 3% with ingestion of the degraded cell contents (~2 min per insect, WDI). This means that ingesting nutrients from seeds needs food preparation, different from vascular tissues, where nutrients are ready for ingestion (*E. heros* ingested xylem sap for a long time > 100 min per insect). Although *E. heros* spent a much shorter time in ingestion events from seed endosperm compared to vascular tissues, seeds are pockets of storage cells highly concentrated in nutrients (Slansky and Panizzi 1987), whereas, vascular tissues are rich in water and low in nutrient concentration (Taiz and Zeiger 2004).

#### Ingestion Phase: Unknown Feeding Site (Eh4)

Waveform Eh4 was seen at all Ri levels applied, but only in 30% of the bugs recorded; the biological meaning of this waveform is uncertain. In the histological correlation attempted for Eh4, in only one occasion plant tissue section was successfully obtained (stylets near xylem vessels), but this was not clear. Compared to Eh2 (xylem), Eh4 is a shorter waveform, highly regular with a very low amplitude, which suggests a passive ingestion (i.e., small force needed to ingest sap). Thus, it is plausible that Eh4 represents short phloem sap ingestion (~3 min per insect, WDI), since, this vascular tissue presents a high internal hydrostatic pressure, forcing phloem sap to flow out (Taiz and Zeiger 2004).

A similar waveform to Eh4 has been described to another stink bug, *Edessa meditabunda* (F.) (Em3 wave) (Lucini and Panizzi 2016), and to the plataspid *Megacopta cribraria* (F.) (I wave) (closely related to Pentatomidae), on soybean stem (Stubbins et al. 2017). Those waveforms were correlated via histological studies with phloem sap ingestion and they share a very low relative amplitude, as well as, Eh4. For now, *E. meditabunda* was the only stink bug species observed to feed on phloem vessels (Lucini and Panizzi 2016), but this species is known to be primarily a stem-feeder (Silva et al. 2012).

In addition, a small change in the voltage level either between transition of Eh1b to Eh4 or Eh2 to Eh4 (which occurred interspersed) suggests that the stylets were in a different vascular cell, i.e., stink bugs may have moved slightly their stylets to seek another vessel causing this change in voltage. This unclear result should be further investigated to determine the correct biological meaning of this waveform.

#### Interruption Phase: Watery Saliva Injection Into Xylem Vessels (Eh5)

In some recordings, brief interruptions (waveform Eh5) were observed during xylem ingestion (waveform Eh2). Eh5 is similar in appearance and electrical characteristics with the interruption waveform Pg4 observed within xylem ingestion of the pentatomid *P. guildinii* on different structures of soybean plants (Lucini et al. 2016) and other sucking insects (e.g., the sharpshooter *Bucephalogonia xanthophis* (Berg) [Miranda et al. 2009], and the chinch bugs *Blissus* spp. [Backus et al. 2013]). According to Backus et al. (2005a, 2013) those interruptions may represent watery salivation and tasting/testing of xylem cells, which probably occur with *E. heros*; since Eh5 occurred mostly at the beginning of xylem sap ingestion, it may indicate testing of the cells.

In conclusion, our results showed that *E. heros* on soybean pod ingest food from xylem vessels and from seed endosperm. At this last food source, we recorded and identified two distinct waveform subtypes, when most of the time the bug prepared the food (breakage the storage cells) to later ingest the degraded cell contents. *E. heros* uses two feeding strategies on soybean pod. On xylem vessels, the bug uses the salivary sheath feeding, with secretion of a complete salivary sheath, whereas, on seed endosperm it switches and use the cell rupture feeding with secretion of an incomplete salivary sheath. The waveform library produced may be used to develop potential management tactics to control this pest, such as evaluate the effect of tolerant/resistant cultivars and evaluate the effect of selective chemical compounds on its feeding behavior.
